# Geographic Variation in Low-Value Orthopedic Services Among Medicare-Enrolled Veterans

**DOI:** 10.1001/jamanetworkopen.2026.35454

**Published:** 2026-09-21

**Authors:** Sooyeon Song, Steven D. Pizer, Christine A. Yee, Samantha G. Auty

**Affiliations:** 1 Department of Health Law, Policy, and Management, Boston University School of Public Health, Boston, Massachusetts; 2 Boston VA Healthcare System, Boston, Massachusetts

## Abstract

This cross-sectional study examines geographic variation in and correlation between low-value orthopedic services across hospital referral regions in a cohort of veterans enrolled in traditional Medicare.

## Introduction

Low-value services (LVSs), interventions that provide limited or no clinical benefit, are recognized sources of wasteful health care spending and are targeted by federal policy initiatives.^1,2^ Orthopedic care accounts for a substantial proportion of Medicare spending and includes several LVSs recently targeted by the Centers for Medicare & Medicaid Services policies, including procedures subject to prior approval to discourage unnecessary use.^2^ Prior studies documented geographic variation in LVSs as well as patient outcomes and spending associated with such services.^3,4^ However, whether regions with high use of one service also have high use of others within the same specialty remains understudied. This distinction has implications for the design of strategies to reduce LVSs, because cross-service clustering would support broad regional interventions, whereas independent variation would suggest procedure-specific interventions. We examined geographic variation in 5 orthopedic LVSs across hospital referral regions (HRRs) and assessed whether regional rates were correlated across services in a cohort of veterans enrolled in traditional Medicare.

## Methods

In this cross-sectional study, we used 2017 to 2022 Medicare fee-for-service claims to identify veterans aged 65 years or older with continuous Medicare enrollment and at least 1 visit to an orthopedic surgeon. This study followed the Strengthening the Reporting of Observational Studies in Epidemiology (STROBE) reporting guideline for cross-sectional studies. The Boston VA Healthcare System institutional review board deemed the study exempt, and the requirement for informed consent was waived because the study used deidentified administrative data. Using established claims-based algorithms (eTables 1-2 in Supplement 1), we measured 5 orthopedic procedures (vertebroplasty, spinal fusion, back imaging, spinal injection, and knee arthroscopy) ordered or delivered by orthopedic surgeons that are considered low-value in certain clinical situations.^4,5^ For each LVS, we calculated HRR-level rates per 100 enrollees meeting procedure-specific clinical criteria, pooled from 2018 to 2022. We assessed cross-service clustering using enrollee-weighted Pearson correlations across HRRs. We interpreted *r*^2^ as the proportion of regional variation in one service explained by variation in another. We estimated 95% CIs using cluster bootstrap resampling at the HRR level. To assess whether measurement error in rarer services attenuated the observed correlations, we computed reliability-corrected correlations. In a sensitivity analysis, we recomputed the correlations using alternative weights (unweighted and eligible-denominator-weighted). Analysis was conducted using Stata 19.5 (StataCorp) between December 2025 and July 2026. Additional details are in the eMethods in Supplement 1.

## Results

The analysis included 306 HRRs serving 1 589 218 enrollees (mean [SD] age, 74.7 [8.6] years; 1 315 873 male [82.8%] and 273 345 female [17.2%]). The prevalence of orthopedic LVSs exhibited geographic variation (Figure). Southern HRRs often fell above the 75th percentile for back imaging but below the 25th percentile for spinal fusion, while western HRRs showed the reverse. Weighted mean rates per 100 enrollees meeting procedure-specific clinical criteria ranged from 0.3 (0.3) for knee arthroscopies to 44.6 (8.5) for back imaging. Variation was largest for spinal injections, where HRRs at the 75th percentile had rates 3.5 times greater than HRRs at the 25th percentile; variation was smallest for back imaging (1.4-fold difference).

**Figure.**
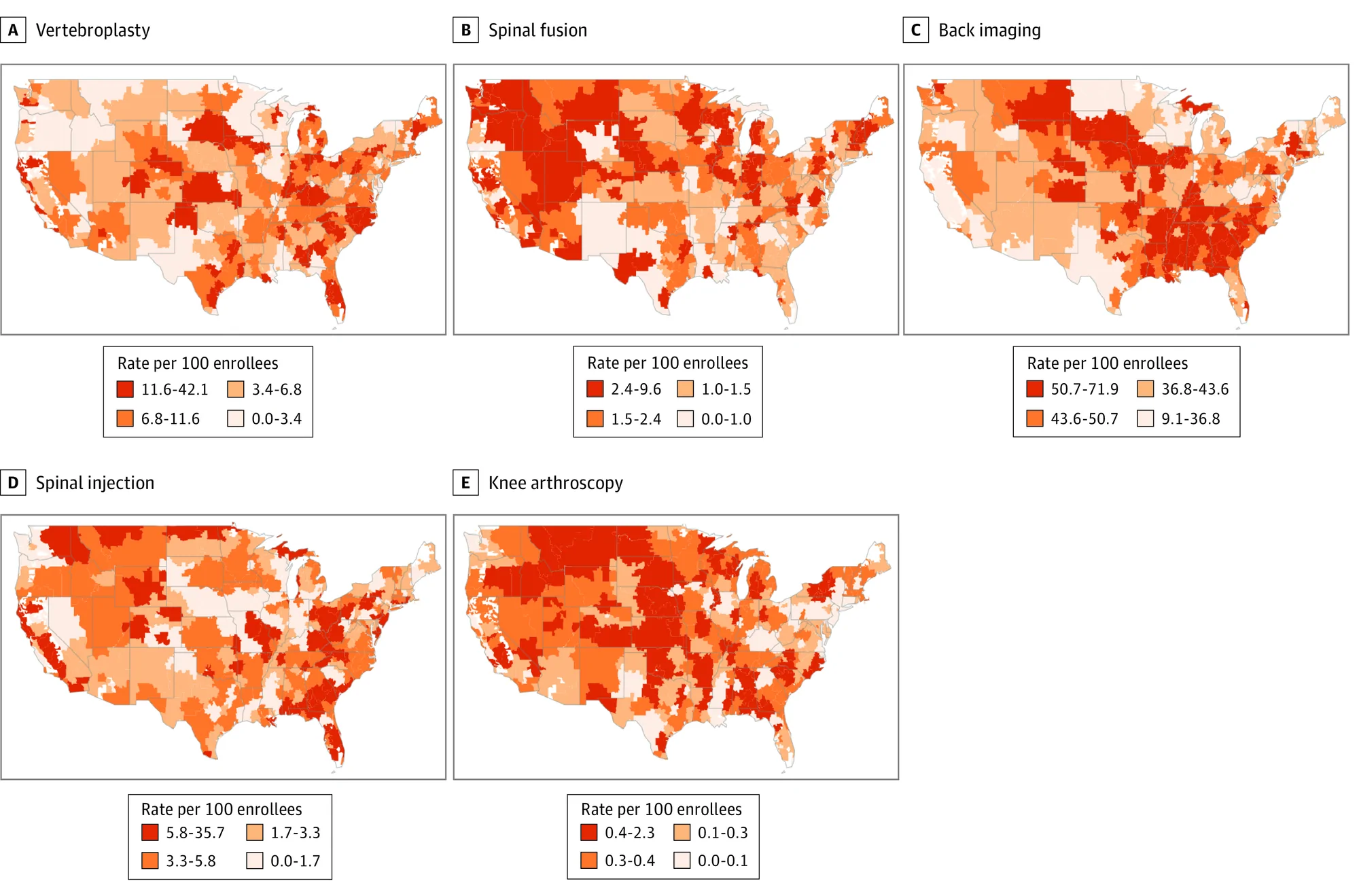
Maps of Geographic Variation in Low-Value Orthopedic Services Among Medicare-Enrolled Veterans, 2018-2022 Rates of 5 low-value orthopedic services per 100 enrollees meeting procedure-specific clinical criteria, pooled over 2018 to 2022, by hospital referral region (HRR). For each service, HRRs are shaded by quartile of the rate distribution, with darker shading indicating higher rates.

Pairwise correlations between orthopedic LVSs were consistently low across services (Table). Specifically, observed correlations ranged from an *r* of −0.21 (95% CI, −0.32 to −0.11) to 0.20 (95% CI, 0.03 to 0.36), with all *r*^2^ being 0.05 or less. Only 12 of 77 HRRs above the 75th percentile for spinal fusion (15.6%) were also above the 75th percentile for back imaging, below the 25% expected under independence. This weak clustering persisted after correcting for measurement error (*r* = −0.24; 95% CI, −0.38 to −0.06) and under alternative weighting (*r* = −0.25; 95% CI, −0.37 to −0.13) (Table).

**Table.** Correlation Matrix of Low-Value Orthopedic Services

Service^b^		*r* (95% CI)^a^				
		Vertebroplasty	Spinal fusion	Back imaging	Spinal injection	Knee arthroscopy
Vertebroplasty						
Observed		1.00	NA^c^	NA^c^	NA^c^	NA^c^
Corrected		1.00	NA^c^	NA^c^	NA^c^	NA^c^
Spinal fusion						
Observed		−0.04 (−0.17 to 0.10)	1.00	NA^c^	NA^c^	NA^c^
Corrected		−0.04 (−0.21 to 0.12)	1.00	NA^c^	NA^c^	NA^c^
Back imaging						
Observed		0.11 (−0.02 to 0.24)	−0.21 (−0.32 to −0.11)	1.00	NA^c^	NA^c^
Corrected		0.13 (−0.02 to 0.29)	−0.25 (−0.37 to −0.13)	1.00	NA^c^	NA^c^
Spinal injection						
Observed		0.20 (0.03 to 0.36)	−0.19 (−0.33 to −0.00)	0.10 (−0.04 to 0.23)	1.00	NA^c^
Corrected		0.22 (0.03 to 0.41)	−0.21 (−0.37 to 0.00)	0.11 (−0.04 to 0.24)	1.00	NA^c^
Knee arthroscopy						
Observed		0.01 (−0.14 to 0.15)	0.08 (−0.01 to 0.17)	0.14 (0.00 to 0.27)	0.03 (−0.09 to 0.18)	1.00
Corrected		0.01 (−0.16 to 0.18)	0.09 (−0.02 to 0.20)	0.16 (0.00 to 0.31)	0.04 (−0.10 to 0.20)	1.00

Abbreviation: NA, not applicable.

^a^ Enrollee-weighted Pearson correlation coefficients of HRR-level rates pooled over 2018 to 2022, across 306 hospital referral regions (HRRs). The 95% CIs were estimated using HRR-level cluster bootstrap resampling with 1000 replications.

^b^ Observed correlations are reported first. Corrected correlations are corrected for attenuation due to measurement error (eMethods in Supplement 1).

^c^ NA because each pair of services is reported once, in the lower half of the matrix.

## Discussion

In this cross-sectional study, each orthopedic LVS showed substantial geographic variation across HRRs among Medicare-enrolled veterans, but rates across different services were weakly correlated. High-use regions for one LVS were not consistently high-use regions for others, complementing prior research^5,6^ by suggesting that regional variation in individual orthopedic LVSs may be largely procedure-specific rather than driven by regional utilization tendencies.

This study has several limitations. First, our findings pertain to veterans whose services were covered under traditional Medicare and may not generalize to nonveteran populations. Second, this study did not investigate underlying factors of geographic variation in LVS frequency. Third, because HRRs approximate referral markets for surgical care, the effective market for outpatient services (eg, back imaging) may be smaller than an HRR, so their correlations may differ from those observed at the HRR level.

These findings may inform procedure-specific strategies for reducing orthopedic LVS, such as prior authorization or insurance network design based on procedure-specific utilization. Further research is needed to examine how procedure-specific patterns of orthopedic LVS translate into differences in overall spending and clinical outcomes to prioritize more targeted policy interventions.

## Notes

Supplement 1. eTable 1. Definitions of Low-Value Orthopedic Services eTable 2. Claims-Based Algorithms Used to Identify Low-Value Orthopedic Services eMethods. eReferences

Supplement 2. Data Sharing Statement

## References

[1] Shrank WH, Rogstad TL, Parekh N. Waste in the US health care system: estimated costs and potential for savings. JAMA. 2019;322(15):1501-1509. doi:10.1001/jama.2019.13978 31589283

[2] Centers for Medicare & Medicaid Services. Wasteful and inappropriate service reduction (WISeR) model. Updated August 4, 2026. Accessed August 11, 2026. https://www.cms.gov/priorities/innovation/innovation-models/wiser

[3] Skinner J. Causes and consequences of regional variations in health care. In: Pauly MV, McGuire TG, Barros PP, eds. Handbook of Health Economics. Vol 2. Elsevier; 2011:45-93. doi:10.1016/B978-0-444-53592-4.00002-5

[4] Chalmers K, Gopinath V, Brownlee S, Saini V, Elshaug AG. Adverse events and hospital-acquired conditions associated with potential low-value care in Medicare beneficiaries. JAMA Health Forum. 2021;2(7):e211719. doi:10.1001/jamahealthforum.2021.1719 35977201 PMC8796970

[5] Schwartz AL, Landon BE, Elshaug AG, Chernew ME, McWilliams JM. Measuring low-value care in Medicare. JAMA Intern Med. 2014;174(7):1067-1076. doi:10.1001/jamainternmed.2014.1541 24819824 PMC4241845

[6] Song Z, Kannan S, Gambrel RJ, . Physician practice pattern variations in common clinical scenarios within 5 US metropolitan areas. JAMA Health Forum. 2022;3(1):e214698. doi:10.1001/jamahealthforum.2021.4698 35977237 PMC8903123

