# Supplementary material for: Geographic Variation in Low-Value Orthopedic Services Among Medicare-Enrolled Veterans
Source: JAMA Netw Open. 2026 Sep 21;9(9):e2635454. doi: 10.1001/jamanetworkopen.2026.35454 (PMC13595179; doi:10.1001/jamanetworkopen.2026.35454)
Supplement: Supplementary file 1 [file jamanetwopen-e2635454-s001.pdf]

# Supplemental Online Content

Song S, Pizer SD, Yee CA, Auty SG. Geographic variation in low-value orthopedic services for medicare-enrolled veterans. *JAMA Netw. Open*. 2026;9(9):e2635454.
doi:10.1001/jamanetworkopen.2026.35454

**eTable 1.** Definitions of Low-Value Orthopedic Services

**eTable 2.** Claims-Based Algorithms Used to Identify Low-Value Orthopedic Services

**eMethods**

**eReferences**

This supplemental material has been provided by the authors to give readers additional information about their work.

© 2026 Song S et al. *JAMA Network Open*.

**eTable 1. Definitions of Low-Value Orthopedic Services**

| **Low-Value Services** | **Eligible Population** (Denominator) | **Detection Criteria** (Numerator) | **Source** |
|---|---|---|---|
| Vertebroplasty for osteoporotic fracture[1] | Patients with vertebral fracture, with osteoporosis diagnosis | Vertebroplasty for osteoporotic vertebral fracture | Chalmers et al., 2021 |
| Arthroscopic surgery for knee osteoarthritis[1-4] | Patients with knee osteoarthritis, without a prior meniscal tear | Arthroscopic knee debridement for knee osteoarthritis or chondromalacia | Schwartz et al., 2023; Radomski et al., 2022; Chalmers et al., 2021; Kim et al., 2025 |
| Spinal fusion for nonspecific low back pain[1,2] | Patients with low back pain, without radicular conditions, herniated disc, scoliosis, or ≥2 radiculopathy diagnoses | Lumbar spinal fusion for nonspecific low back pain | Chalmers et al., 2021; Kim et al., 2025 |
| Spinal injections for low back pain[2-5] | Patients with a diagnosis of low back pain, without radiculopathy, and without spinal injection during or within 14 days after an inpatient stay | Spinal injection for low back pain in the outpatient setting, not associated with an inpatient stay | Sanghavi et al., 2021; Schwartz et al., 2023; Radomski et al., 2022; Kim et al., 2025 |
| Back imaging for nonspecific low back pain[2-5] | Patients with a first diagnosis of back pain, without exclusion conditions, and without back imaging >6 weeks after the first diagnosis | Back imaging received within 6 weeks of the first diagnosis of back pain | Sanghavi et al., 2021; Schwartz et al., 2023; Radomski et al., 2022; Kim et al., 2025 |

© 2026 Song S et al. *JAMA Network Open*.

**eTable 2. Claims-Based Algorithms Used to Identify Low-Value Orthopedic Services**

| Low-Value Service | CPT/HCPCS Codes used to identify health service use | ICD-10 Codes used to identify patients with a medical condition qualifying them to receive each service | ICD-10 and other codes used to exclude patients when applying the specific criteria |
|---|---|---|---|
| Vertebroplasty for osteoporotic fracture | 22510 22511 22512 22513 22514 22515 | M8000 M8000XA M8000XD M8000XG M8000XK M8000XP M8000XS M8008 M8008XA M8008XD M8008XG M8008XK M8008XP M8008XS M8080 M8080XA M8080XD M8080XG M8080XK M8080XP M8080XS M8088 M8088XA M8088XD M8088XG M8088XK M8088XP M8088XS | C412 C7B03 C7951 C7952 C900 C9000 C9001 C9002 D166 D1809 D47Z9 D480 D492 |
| Arthroscopic surgery for knee osteoarthritis | 29877 29879 29880 29881 G0289 | M224 M942 M150 M153 M154 M158 M159 M17 M199 | S832 M232 M2576 |
| Spinal fusion for nonspecific low back pain | 0275T 22533 22534 22558 22630 63005 63012 63017 63030 63035 63042 63047 63173 63185 63190 63191 63200 63267 63272 | M4720 M4726 M4727 M4728 M47816 M47817 M47818 M47819 M47896 M47897 M47898 M47899 M479 M4806 M4807 M4808 M5116 M5117 M5126 M5127 M5136 M5137 M5186 M5187 M519 M532X6 M532X7 M532X8 M533 M5386 M5387 M5388 M5416 M5417 M5418 M5430 M5431 M5432 M5440 M5441 M5442 M545 M5489 M549 M9903 M9904 M9923 M9933 M9943 M9953 M9963 M9973 M9983 M9984 S335 S336 S338 S339 S39002 S39012 S39092 S3982 S3992 | M41 M410 M4100 M4102 M4103 M4104 M4105 M4106 M4107 M4108 M411 M4111 M41112 M41113 M41114 M41115 M41116 M41117 M41119 M4112 M41122 M41123 M41124 M41125 M41126 M41127 M41129 M412 M4120 M4122 M4123 M4124 M4125 M4126 M4127 M413 M4130 M4134 M4135 M414 M4140 M4141 M4142 M4143 M4144 M4145 M4146 M4147 M415 M4150 M4152 M4153 M4154 M4155 M4156 M4157 M418 M4180 M4182 M4183 M4184 M4185 M4186 M4187 M419 M464 M4640 |

© 2026 Song S et al. *JAMA Network Open*.

| Low-Value Service | CPT/HCPCS Codes used to identify health service use | ICD-10 Codes used to identify patients with a medical condition qualifying them to receive each service | ICD-10 and other codes used to exclude patients when applying the specific criteria |
|---|---|---|---|
| | | | M4641 M4642 M4643 M4644 M4645 M4646 M4647 M4648 M4649 M472 M4720 M4721 M4722 M4723 M4724 M4725 M4726 M4727 M4728 M510 M5104 M5105 M5106 M511 M5114 M5115 M5116 M5117 M512 M5124 M5125 M5126 M5127 M514 M5144 M5145 M5146 M5147 M518 M5184 M5185 M5186 M5187 M519 M961 |
| Spinal injections for low back pain | 62311 62322 62323 64483 20552 20553 64493 64475 J1438 | M4720 M4726 M4727 M4728 M47816 M47817 M47818 M47819 M47896 M47897 M47898 M47899 M479 M4806 M4807 M4808 M5116 M5117 M5126 M5127 M5136 M5137 M5186 M5187 M519 M532X6 M532X7 M532X8 M533 M5386 M5387 M5388 M5416 M5417 M5418 M5430 M5431 M5432 M5440 M5441 M5442 M545 M5489 M549 M9903 M9904 M9923 M9933 M9943 M9953 M9963 M9973 M9983 M9984 S335 S336 S338 S339 S39002 S39012 S39092 S3982 S3992 | M4720 M4726 M4727 M4728 M5116 M5117 M5416 |
| Back imaging for nonspecific low back pain | 72010 72020 72052 72100 72110 72114 72120 72200 72202 72220 72131 72133 | M4720 M4726 M4727 M4728 M47816 M47817 M47818 M47819 M47896 M47897 | C D0 D37-D44 D46 D47 D48 D49 Z85 S0 S10 S11 S12 S130 S131 S132 S140 S1411 S1412 |

© 2026 Song S et al. *JAMA Network Open*.

| Low-Value Service | CPT/HCPCS Codes used to identify health service use | ICD-10 Codes used to identify patients with a medical condition qualifying them to receive each service | ICD-10 and other codes used to exclude patients when applying the specific criteria |
|---|---|---|---|
| | 72141 72142 72146 72149 72156 72157 72158 | M47898 M47899 M479 M4806 M4807 M4808 M5116 M5117 M5126 M5127 M5136 M5137 M5186 M5187 M519 M532X6 M532X7 M532X8 M533 M5386 M5387 M5388 M5416 M5417 M5418 M5430 M5431 M5432 M5440 M5441 M5442 M545 M5489 M549 M9903 M9904 M9923 M9933 M9943 M9953 M9963 M9973 M9983 M9984 S335 S336 S338 S339 S39002 S39012 S39092 S3982 S3992 | S1413 S1414 S1415 S21 S22 S230 S231 S232 S240 S241 S26 S27 S28 S310 S32 S330 S331 S332 S333 S334 S340 S3411 S3412 S3413 S36 S37 S38 S42 S430 S431 S432 S433 S490 S491 S52 S530 S531 S532 S533 S57 S58 S590 S591 S592 S62 S630 S631 S632 S633 S634 S72 S730 S77 S78 S790 S791 S82 S830 S831 S87 S88 S890 S891 S892 S893 S92 S930 S931 S97 S98 S990 S991 S992 T79 A15 A17 A18 A19 F11 F13 F14 F15 M462 M463 M464 M465 G061 G834 I33 I38 I39 A40 A41 R50 R6883 R630 R634 R636 R538 R61 D649 |

Note: CPT indicates Current Procedural Terminology; HCPCS, Healthcare Common Procedure Coding System; ICD-10, International Classification of Diseases, Tenth Revision. Code stems (eg, C, D0, Z85) include all codes beginning with those characters, and ranges (eg, D37-D44) include all codes within the range.

© 2026 Song S et al. *JAMA Network Open*.

**eMethods**

**Data and Sample.** We used 2017-2022 Medicare fee-for-service (FFS) professional, hospital outpatient, and Medicare Provider Analysis and Review claims, with 2017 serving as a look-back year. The study period was 2018-2022. Our Medicare FFS claims data were linked to Veterans Health Administration enrollment files, restricting the cohort to Veteran enrollees.

We included all eligible Medicare-enrolled Veterans across all 306 hospital referral regions (HRRs). Enrollees met standard Medicare cohort criteria, including age ≥65 years, continuous Parts A and B enrollment, and exclusion of end-stage renal disease and Medicare Advantage enrollment. We included enrollees with at least 1 professional claim from an orthopedic surgeon, defined using Centers for Medicare & Medicaid Services specialty code 20 in the Medicare Data on Provider Practice and Specialty file. The unit of analysis was the HRR, with rates pooled from 2018-2022.

**Variables.** We defined five low-value orthopedic services (LVS). The five services were vertebroplasty, spinal fusion, back imaging, spinal injection, and knee arthroscopy, identified using conservative, established claims-based algorithms (eTables 1-2). Services were selected if prior studies identified them as commonly provided by specialists (orthopedists),[6,7] or if orthopedic surgeons ordered or delivered >10% of the service in our data.

For each service, denominators were diagnosis-defined eligible enrollees among Veterans with at least 1 orthopedic surgeon visit in that year. Because LVS are defined as interventions that provide limited or no clinical benefit across patient subgroups, we focused on unadjusted regional rates to characterize absolute geographic variation in potentially avoidable volume.

We identified LVS occurrences at the enrollee level. When an enrollee received the same LVS multiple times, each occurrence was counted as a separate incidence. Repeat services occurring within 7 days were collapsed into a single episode to avoid double counting of the same clinical encounter, consistent with prior literature.[5,8] The 7-day window collapses claims that represent components of a single procedure billed on separate dates (eg, the professional and technical components of an imaging study).

We restricted analyses to services ordered or delivered by orthopedic surgeons to focus on specialty-specific practice patterns. This restriction reduces confounding by specialty mix, as some procedures are also performed by neurosurgeons and pain medicine specialists.

**Statistical Analysis.** We used 306 hospital referral regions defined by the Dartmouth Atlas, the standard set of health care market units in the US, as the unit of geographic analysis. For each LVS, we calculated HRR-level rates pooled over 2018-2022 (n=306 HRRs).

For geographic maps, we displayed HRR-level rates pooled over 2018-2022 calculated as total episodes divided by total eligible enrollees in each HRR. We described geographic variation using the ratio of the 75th to 25th percentile, which is more robust to extreme values than the 90th to 10th percentile ratio. There were no missing data in the analytic variables, as all measures were derived from complete Medicare claims. Hospital referral regions with zero observed use of a service (11% of HRRs for vertebroplasty) represented true zeros and were retained as observed values rather than imputed.

We computed pairwise Pearson correlation coefficients across pooled HRR-level rates, weighted by the total number of enrollees in each HRR to account for differences in estimate

© 2026 Song S et al. *JAMA Network Open*.

precision. To account for within-region correlation, we estimated 95% CIs by resampling HRRs with replacement, with 1,000 replications. We additionally examined cross-service overlap by calculating the proportion of HRRs above the 75th percentile for one service that were also above the 75th percentile for another.

To assess whether measurement error attenuated the observed correlations, we computed reliability-corrected correlations. We estimated the reliability of each service's annual HRR-level rate as its mean cross-year correlation, which reflects reliability under stable regional patterns and independent annual noise. We converted annual reliabilities to 5-year equivalents to match the pooled rates. We then divided each pairwise correlation by the geometric mean of the two services' reliabilities. The Table presents both observed and corrected correlations.

In a sensitivity analysis, we recomputed the correlations using alternative weights. Unweighted correlations assign equal weight to each HRR, making the region, rather than the enrollee, the unit of inference. Eligible-denominator-weighted correlations weight each pair of services by the harmonic mean of their eligible denominators, which is proportional to the precision of the pair of rates being correlated.

© 2026 Song S et al. *JAMA Network Open*.

**eReferences**


1. Chalmers K, Gopinath V, Brownlee S, Saini V, Elshaug AG. Adverse events and hospital-acquired conditions associated with potential low-value care in Medicare beneficiaries. *JAMA Health Forum*. 2021;2(7):e211719. doi:10.1001/jamahealthforum.2021.1719

2. Kim DD, Fendrick AM. Projected savings from reducing low-value services in Medicare. *JAMA Health Forum*. 2025;6(8):e253050. doi:10.1001/jamahealthforum.2025.3050

3. Schwartz AL, Zhao X, Sileanu FE, et al. Variation in low-value service use across Veterans Affairs facilities. *J Gen Intern Med.* 2023;38(10):2245-2253. doi:10.1007/s11606-023-08157-9

4. Radomski TR, Zhao X, Lovelace EZ, et al. Use and cost of low-value health services delivered or paid for by the Veterans Health Administration. *JAMA Intern Med.* 2022;182(8):832-839. doi:10.1001/jamainternmed.2022.2482

5. Sanghavi P, McWilliams JM, Schwartz AL, Zaslavsky AM. Association of low-value care exposure with health care experience ratings among patient panels. *JAMA Intern Med.* 2021;181(7):941-948. doi:10.1001/jamainternmed.2021.1974

6. Song Z, Kannan S, Gambrel RJ, et al. Physician practice pattern variations in common clinical scenarios within 5 US metropolitan areas. *JAMA Health Forum*. 2022;3(1):e214698. doi:10.1001/jamahealthforum.2021.4698

7. Schwartz AL, Jena AB, Zaslavsky AM, McWilliams JM. Analysis of physician variation in provision of low-value services. *JAMA Intern Med.* 2019;179(1):16-25. doi:10.1001/jamainternmed.2018.5086

8. Schwartz AL, Landon BE, Elshaug AG, Chernew ME, McWilliams JM. Measuring low-value care in Medicare. JAMA Intern Med. 2014;174(7):1067-1076. doi:10.1001/jamainternmed.2014.1541


© 2026 Song S et al. *JAMA Network Open*.
