# Supplementary material for: Geographic Variation in Low-Value Orthopedic Services Among Medicare-Enrolled Veterans
Source: JAMA Netw Open. 2026 Sep 21;9(9):e2635454. doi: 10.1001/jamanetworkopen.2026.35454 (PMC13595179; doi:10.1001/jamanetworkopen.2026.35454)
Supplement: Supplementary file 2 [file jamanetwopen-e2635454-s002.pdf]

# Data Sharing Statement

Song. Geographic Variation in Low-Value Orthopedic Services Among Medicare-Enrolled Veterans. *JAMA Netw Open*. Published September 22, 2026.
doi:10.1001/jamanetworkopen.2026.35454

**Data**
**Data available:** No

**Additional Information**
**Explanation for why data not available:** The data used in this study are Medicare fee-for-service claims obtained through a Data Use Agreement with the Centers for Medicare & Medicaid Services, which prohibits redistribution of the data to third parties.
